# A Retrospective Study of the Impact of 21-Gene Recurrence Score Assay on Treatment Choice in Node Positive Micrometastatic Breast Cancer

**DOI:** 10.3390/ph8010107

**Published:** 2015-03-17

**Authors:** Thomas G. Frazier, Kevin R. Fox, J. Stanley Smith, Christine Laronga, Anita McSwain, Devchand Paul, Michael Schultz, Joseph Stilwill, Christine Teal, Tracey Weisberg, Judith F. Vacchino, Amy P. Sing, Dasha Cherepanov, Wendy Hsiao, Eunice Chang, Michael S. Broder

**Affiliations:** 1 Main Line HealthCare Breast Surgical Specialists, Susquehanna Bank Building, 101 South Bryn Mawr Ave, Suite 201, Bryn Mawr, PA 19010, USA; E-Mail: tgf@tgfsurgery.com; 2 Rena Rowan Breast Center at the Abramson Cancer Center of the University of Pennsylvania, 3400 Civic Center Boulevard, 3 West Pavilion, Philadelphia, PA 19104, USA; E-Mail: Kevin.Fox@uphs.upenn.edu; 3 Penn State Hershey Breast Center, Penn State College of Medicine, 30 Hope Drive, MC EC008, P.O. Box 859, Hershey, Pennsylvania 17033, USA; E-Mail: jsmith9@hmc.psu.edu; 4 H. Lee Moffitt Cancer Center and Research Institute, 12902 Magnolia Drive, Tampa, FL 33612, USA; E-Mail: Christine.Laronga@moffitt.org; 5 The George Washington University, GW Medical Faculty Associates, 2300 M Street, NW 8th floor, DC Level, Washington, DC 20037, USA; E-Mails: amcswain@mfa.gwu.edu (A.M.); cteal@mfa.gwu.edu (C.T.); 6 Rocky Mountain Cancer Centers, 4700 East Hale Parkway, Suite 400, Denver, CO 80220, USA; E-Mail: devchand.paul@usoncology.com; 7 University of Maryland St. Joseph Medical Center, 7601 Osler Drive, Towson, MD 21204, USA; E-Mail: michaelschultz@umm.edu; 8 Midwest Cancer Care Physicians at the Menorah Medical Center, 2316 East Meyer Boulevard, 1 West, Kansas City, MO 64132, USA; E-Mail: Joseph.stilwill@hcahealthcare.com; 9 The Maine Center for Cancer Medicine, 100 Campus Drive, unit 108, Scarborough, Maine 04074, USA; E-Mail: weisbt@mccm.org; 10 Genomic Health, Inc., 301 Penobscot Drive, Redwood City, CA 94063, USA; E-Mails: JVacchino@genomichealth.com (J.F.V.); asing@genomichealth.com (A.P.S.); 11 Partnership for Health Analytic Research, LLC, 280 S. Beverly Drive, Suite 404, Beverly Hills, CA 90212, USA; E-Mails: echang@pharllc.com (E.C.); mbroder@pharllc.com (M.S.B.); 12 Keck School of Medicine, University of Southern California, Los Angeles, CA 90089, USA; E-Mail: wendy.hsiao@usc.edu

**Keywords:** clinical utility, genomics, recurrence risk, chemotherapy, breast cancer

## Abstract

To assess clinical utility of the 21-gene assay (Onco*type* DX^®^ Recurrence Score^®^), we determined whether women with HER2(−)/ER+ pN1mi breast cancer with low (<18) Recurrence Scores results are given adjuvant chemotherapy in a lower proportion than those with high scores (≥31). This was a multicenter chart review of ≥18 year old women with pN1mi breast cancer, HER2(−)/ER+ tumors, ductal/lobular/mixed histology, with the assay ordered on or after 1 January 2007. One hundred and eighty one patients had a mean age of 60.7 years; 82.9% had ECOG performance status 0; 33.7% had hypertension, 22.7% had osteoporosis, 18.8% had osteoarthritis, and 8.8% had type-2 diabetes. Mean Recurrence Score was 17.8 (range: 0–50). 48.6% had a mastectomy; 55.8% had a lumpectomy. 19.8% of low-risk group patients were recommended chemotherapy *vs.* 57.9% in the intermediate-risk group and 100% in the high-risk group (*p* < 0.001). A total of 80.2% of the low-risk group were recommended endocrine therapy alone, while 77.8% of the high-risk group were recommended both endocrine and chemotherapy (*p* < 0.001). The Onco*type* DX Recurrence Score result provides actionable information that can be incorporated into treatment planning for women with HER2(−)/ER+ pN1mi breast cancer. The Recurrence Score result has clinical utility in treatment planning for HER2(−)/ER+ pN1mi breast cancer patients.

## 1. Introduction

In 2015, more than 231,840 women in the United States will be diagnosed with invasive breast cancer and almost 40,290 will die from it, making breast cancer the most common cancer diagnosis among American women, as well as the second leading cause of cancer death in women [1]. Advances in breast cancer diagnostics and in histopathological and molecular analysis techniques have resulted in an increase in the number of women diagnosed with micrometastatic (pN1mi) breast cancer (≤2 mm axillary node metastasis) [2,3,4]. Studies report conflicting results regarding the clinical significance and implications of these micrometastases, with some data suggesting they do not confirm increased risk for distant recurrence and other data suggesting that they do [4].

For women with disease that is human epidermal growth factor receptor 2 (HER2)-negative, estrogen receptor (ER) positive (HER2[−]/ER+) and who have micrometastasis, clinical practice varies, with some women receiving adjuvant chemotherapy and others prescribed endocrine therapy alone [5]. Node-negative patients treated with tamoxifen alone have a 10-year recurrence rate of 15%, suggesting that many women with pN1mi/HER2(−)/ER+ disease could forgo chemotherapy, if those at lowest risk could be properly identified [6].

The 21-gene assay (Onco*type* DX [Genomic Health, Inc., Redwood City, CA, USA]) for invasive breast cancer, employs reverse transcriptase polymerase chain reaction (RT-PCR) to measure the expression of 16 breast cancer recurrence-related genes and five reference genes from paraffin-embedded tumor tissue. From this expression profile, a Recurrence Score result ranging from 0 to 100 is calculated and stratified into low recurrence risk (<18), intermediate recurrence risk (18–30), and high recurrence risk (≥31) groups [7]. The prognostic and predictive value of the assay has been previously demonstrated in node-negative disease [7,8]. A more recent study using tumor samples from patients enrolled in the Southwest Oncology Group-8814 trial also demonstrated the assay’s prognostic and predictive value in the node-positive population [9].

The test is being used on women with micrometastatic breast cancer, but it is not known whether the Recurrence Score result is useful in guiding clinical decisions beyond standard histopathologic characteristics. “Clinical utility” describes the ability of a test result to influence patient management and, according to the National Comprehensive Cancer Network, must be established for a genomic diagnostic assay to be useful in routine clinical practice [10]. To address this gap, we conducted a multicenter, medical record review study to determine whether women with HER2(−)/ER+ pN1mi breast cancer who have low (<18) Recurrence Score results are given adjuvant chemotherapy in a lower proportion than those with high Recurrence Score result (≥31).

## 2. Materials and Methods

### 2.1. Study Design and Setting

This study collected data using medical record review and included women with HER2(−)/ER+ micrometastatic breast cancer treated at nine breast cancer centers in the United States. The sites were selected based on high volume of pN1mi breast cancer patients treated and to reflect a wide variety of practice settings and geographic locations. No patient identifiers were collected, and the study was granted a waiver of informed consent by the Institutional Review Boards at each institution.

### 2.2. Patient Population

At each of the nine centers, trained abstractors reviewed the medical records of female patients ≥18 years with HER2(−)/ER+ pN1mi breast cancer; ductal, lobular, or mixed histology; and an Onco*type* DX Recurrence Score test ordered between January 2007 through December 2012. Patients were excluded if they were diagnosed with synchronous contralateral invasive breast cancer, had tubular/colloid or metaplastic histology, had an adjuvant chemotherapy recommendation before the Onco*type* DX Recurrence Score test was ordered, or had more than one Onco*type* DX Recurrence Score result.

### 2.3. Data

Data included patient characteristics, breast cancer surgery type, pathologic data, comorbidities, Onco*type* DX results, and systemic breast cancer treatment recommended post-Recurrence Score result. The primary aim was to determine whether chemotherapy was recommended in lower proportion in HER2(−)/ER+ pN1mi breast cancer women with lower Recurrence Score results. The medical record abstraction form was designed in conjunction with physician experts (KRF, TGF, JSS). To ensure consistent data abstraction across sites, all abstractors received training in correctly applying inclusion/exclusion criteria and entering data. Data were collected using a secure, web-based application. Regular data quality assurance included checks for content, inconsistencies, and missing fields. De-identified data from each site were then combined into a single database. To allow us to compare our sample to the larger group of patients on whom the assay was ordered, Genomic Health, Inc. provided aggregate data on all pN1mi patients tested nationwide during the same timeframe.

### 2.4. Statistical Analysis

An a priori power calculation was performed. The goal was to have 80% power to detect a 40% difference in a recommendation for adjuvant chemotherapy between high and low Recurrence Score results using a one-sided test at a significance level of 0.05. The 40% difference was based on the assumption that 90% of high Recurrence Score result patients would receive adjuvant chemotherapy, compared to 50% of low score patients. The distribution of Recurrence Score results among patients tested was assumed approximately 10% high, 30% intermediate, and 60% low risk, based on distribution of scores in the Genomic Health, Inc. commercial database. Based on these assumptions, 175 patient records were required to appropriately power the study.

All analyses were done in a pooled database, which combined data from each study site, since individual sites had too few patients for site-based analyses or comparisons across study sites. Descriptive statistics were reported by Recurrence Score result categories (*i.e.*, <18 [low risk], 18–30, *vs.* ≥31 [high risk]). Adjuvant treatment recommendations were summarized as the proportion of patients who received the particular treatment recommendation (endocrine therapy, chemotherapy, endocrine and chemotherapy combination, specific types of chemotherapy regimens), and these summary statistics were presented by the Recurrence Score risk categories. To compare across Recurrence Score groups, Chi-square and F-tests were used for categorical and continuous variables, respectively. For categorical variables, when some cell counts were less than five, exact Pearson Chi-square test was used. To compare treatment recommendations, statistical testing was performed both to compare across all groups and between the low and high Recurrence Score groups. To determine the extent to which our study sample was similar to the broader population of patients tested, we compared the distribution of select variables (e.g., patient characteristics, tumor histopathologic characteristics, and Recurrence Score results) observed in this study sample with those observed in all pN1mi patients in the Genomic Health, Inc. database who had the Onco*type* DX test. Missing data were not imputed; counts of missing responses for variables were reported. All statistical analyses were performed using SAS^®^ version 9.3 (SAS Institute, Cary, NC, USA).

## 3. Results and Discussion

Medical records for 218 patients were reviewed. Thirty-seven ineligible records were excluded: 17 did not have micrometastatic disease, 7 did not fall within the required date range, 6 had synchronous contralateral invasive cancer, and 7 for a variety of other reasons (e.g., more than one Onco*type* DX test, inadequate data in the chart to complete abstraction, or left care at the site). Data abstraction was completed for 181 female patients with HER2(−)/ER+ pN1mi breast cancer. Overall, the mean Recurrence Score result was 17.8 (range: 0–50; median: 16), with 58.6% (*n* = 106) of patients falling in the low score group (<18), 31.5% (*n* = 57) in the intermediate score group (18–30), and 9.9% (*n* = 18) in the high score group (≥31). Mean patient age was 60.7 years (range: 34–83), and 75.1% were postmenopausal (Table 1). Most patients had an ECOG performance status of 0 or 1 (82.9% with 0 and 15.5% with 1). The most common comorbidities were hypertension (33.7%), osteoporosis/osteopenia (22.7%), osteoarthritis (18.8%), type 2 diabetes (8.8%), and chronic obstructive pulmonary disease (5.0%). Only the prevalence of coronary artery disease was there noted to be a statistically significant difference at baseline between patients in different Recurrence Score risk categories (0.9% in Recurrence Score <18, 3.5% in 18–30, and 16.7% in ≥31; *p* = 0.009) (Table 1).

Lumpectomy was the most common treatment, performed on 55.8% of patients compared to 48.6% women having mastectomy (Table 2). Overall, sentinel lymph node biopsy without axillary lymph node dissection was performed in 60.2% of patients; 38.1% of women had a sentinel lymph node biopsy with complete axillary node dissection. The proportions with various types of nodal treatment differed between groups. Sentinel lymph node biopsy with concurrent axillary node dissection was the most common procedure performed in the high Recurrence Score group (66.7%), whereas sentinel lymph node biopsy without an axillary node dissection was the most common procedure in the low Recurrence Score group (67.9%) (*p* = 0.041) (Table 2).

The mean tumor size was 1.8 cm (45.9% had tumor size 1.1–2.0 cm; 33.1% had tumors >2 cm). Tumors tended to be larger in the high Recurrence Score group. In patients with a score ≥31, 61.1% (*n* = 11) had tumors >2.0 cm, whereas in the group with a score <18, only 21.7% (*n* = 23) had tumors of that size range (*p* = 0.002). However, for every tumor size category, there was a wide distribution of Recurrence Score results demonstrating that tumor size cannot predict the Recurrence Store result. A single focus of tumor was present in 77% (*n* = 137) of cases, multiple foci in 23% (*n* = 41), and no response was given for three patients. A large majority of patients had tumors with ductal histology (84.5%), 11.0% had lobular, and 4.4% had mixed. Tumor focality and histologic type did not differ significantly across Recurrence Score groups (Table 2).

Nottingham Histologic grade was recorded as 1 in 34.3% of tumors, 2 in 48.1% of cases, and 3 in 17.7%. There were significant differences across the groups, with 61.1% (*n* = 11) of the high Recurrence Score group having grade 3 tumors, compared to 9.4% (*n* = 10) in the low score group (*p* < 0.001). Results show that for any histologic grade, there was a wide distribution of Recurrence Score results indicating that grade alone cannot predict the Recurrence Score result. Lymphovascular invasion was present in 61.1% (*n* = 11) of the tumors in the high Recurrence Score group, compared to 17.0% (*n* = 18) in the low score group (*p* = 0.010). All groups had 0% that tested positive for HER2, and all tumors were ER+ by IHC. In the high score group, 27.8% (*n* = 5) were PR positive, compared to 99.1% (*n* = 105) in the low score group (*p* < 0.001) (Table 2).

**Table 1:** Patient characteristics and medical history by Recurrence Score result category.

	Recurrence Score <18 *n* = 106; 58.6%		Recurrence Score 18–30 *n* = 57; 31.5%		Recurrence Score ≥31 *n* = 18; 9.9%		Total *n* = 181		*p* value ^a^
	Number	Estimate	Number	Estimate	Number	Estimate	Number	Estimate	
**Age (years)** , mean (SD) [median; range]	106	61.7 (10.2) [61; 34–81]	57	58.3 (10.6) [56; 35–83]	18	62.6 (10.2) [62; 39–81]	181	60.7 (10.4) [60; 34–83]	0.099
**Age (years)**, *n* (%)									0.060
<50	12	(11.3)	13	(22.8)	1	(5.6)	26	(14.4)	
50-64	50	(47.2)	30	(52.6)	12	(66.7)	92	(50.8)	
≥65	44	(41.5)	14	(24.6)	5	(27.8)	63	(34.8)	
**ECOG performance status**, *n* (%)									0.685
0: fully active	87	(82.1)	48	(84.2)	15	(83.3)	150	(82.9)	
1: restricted activity	18	(17.0)	8	(14.0)	2	(11.1)	28	(15.5)	
2: capable of all self-care	1	(0.9)	1	(1.8)	1	(5.6)	3	(1.7)	
3: capable of only limited self-care	0	(0.0)	0	(0.0)	0	(0.0)	0	(0.0)	
4: completely disabled	0	(0.0)	0	(0.0)	0	(0.0)	0	(0.0)	
**Postmenopausal ^b^**, *n* (%)									
Yes	84	(79.2)	37	(64.9)	15	(83.3)	136	(75.1)	0.091
**Comorbidity**, *n* (%)									
**Hypertension**	30	(28.3)	22	(38.6)	9	(50.0)	61	(33.7)	0.127
**Osteoporosis/osteopenia**	22	(20.8)	15	(26.3)	4	(22.2)	41	(22.7)	0.720
**Osteoarthritis**	20	(18.9)	11	(19.3)	3	(16.7)	34	(18.8)	0.999
**Type 2 diabetes**	6	(5.7)	8	(14.0)	2	(11.1)	16	(8.8)	0.190
**Chronic obstructive pulmonary disease**	6	(5.7)	2	(3.5)	1	(5.6)	9	(5.0)	0.886
**Valvular heart disease**	6	(5.7)	2	(3.5)	0	(0.0)	8	(4.4)	0.652
**Coronary artery disease**	1	(0.9)	2	(3.5)	3	(16.7)	6	(3.3)	0.009
**Prior invasive breast cancer**	2	(1.9)	2	(3.5)	1	(5.6)	5	(2.8)	0.670
**Congestive heart failure**	0	(0.0)	1	(1.8)	1	(5.6)	2	(1.1)	0.072
**Type 1 diabetes**	0	(0.0)	1	(1.8)	1	(5.6)	2	(1.1)	0.072
**Liver disease**	1	(0.9)	0	(0.0)	1	(5.6)	2	(1.1)	0.287
**Elevated creatinine**	1	(0.9)	0	(0.0)	1	(5.6)	2	(1.1)	0.287
**Chronic kidney disease**	1	(0.9)	0	(0.0)	0	(0.0)	1	(0.6)	0.999

SD (standard deviation); ECOG (Eastern Cooperative Oncology Group); ^a^ *p* < 0.05 for a statistically significant difference across the three Recurrence Score groups; ^b^ Determined by criteria in the NCCN Guidelines 2014.

**Table 2:** Surgical treatment and tumor histopathologic characteristics by Recurrence Score result category.

	Recurrence Score <18 *n* = 106; 58.6%		Recurrence Score 18–30 *n* = 57; 31.5%		Recurrence Score ≥31 *n* = 18; 9.9%		Total *n* = 181		*p* value ^a^
	Number	Estimate	Number	Estimate	Number	Estimate	Number	Estimate	
**Surgical treatment**, *n* (%)									
**Lumpectomy**	57	(53.8)	34	(59.6)	10	(55.6)	101	(55.8)	0.771
**Mastectomy**	52	(49.1)	26	(45.6)	10	(55.6)	88	(48.6)	0.755
**Dissections and biopsies**									0.041
SLNB without ALND	72	(67.9)	31	(54.4)	6	(33.3)	109	(60.2)	
SLNB with ALND	33	(31.1)	24	(42.1)	12	(66.7)	69	(38.1)	
ALND without SLNB	1	(0.9)	2	(3.5)	0	(0.0)	3	(1.7)	
**Tumor size (cm)**, mean (SD) [median; range]	106	1.6 (1.1) [1; 0–6]	57	2.1 (1.2) [2; 1–7]	18	2.3 (0.6) [2; 1–4]	181	1.8 (1.1) [2; 0–7]	0.013
**Tumor size (cm)**, *n* (%)									0.002
≤0.5	9	(8.5)	1	(1.8)	0	(0.0)	10	(5.5)	
0.6–1.0	18	(17.0)	10	(17.5)	0	(0.0)	28	(15.5)	
1.1–2.0	56	(52.8)	20	(35.1)	7	(38.9)	83	(45.9)	
>2.0	23	(21.7)	26	(45.6)	11	(61.1)	60	(33.1)	
**Tumor focality**, *n* (%)									0.277
Number of non-missing	103		57		18		178		
Single focus	75	(72.8)	48	(84.2)	14	(77.8)	137	(77.0)	
Multiple foci	28	(27.2)	9	(15.8)	4	(22.2)	41	(23.0)	
**Histologic type**, *n* (%)									0.184
Ductal	85	(80.2)	50	(87.7)	18	(100.0)	153	(84.5)	
Lobular	14	(13.2)	6	(10.5)	0	(0.0)	20	(11.0)	
Mixed	7	(6.6)	1	(1.8)	0	(0.0)	8	(4.4)	
Metaplastic or tubular/colloid	0	(0.0)	0	(0.0)	0	(0.0)	0	(0.0)	
**Overall histologic grade (Nottingham Histologic Score)**, *n* (%)									<0.001
Grade 1	44	(41.5)	17	(29.8)	1	(5.6)	62	(34.3)	
Grade 2	52	(49.1)	29	(50.9)	6	(33.3)	87	(48.1)	
Grade 3	10	(9.4)	11	(19.3)	11	(61.1)	32	(17.7)	
**Evidence of lymphovascular invasion**, *n* (%)									0.010
Not identified	67	(63.2)	32	(56.1)	5	(27.8)	104	(57.5)	
Present	18	(17.0)	16	(28.1)	11	(61.1)	45	(24.9)	
Indeterminate	5	(4.7)	2	(3.5)	1	(5.6)	8	(4.4)	
Not reported	16	(15.1)	7	(12.3)	1	(5.6)	24	(13.3)	
**HER2 testing by Onco*type* DX HER2 score**, *n* (%)									0.999
Number of non-missing	103		57		18		178		
Negative (<10.7)	102	(99.0)	56	(98.2)	18	(100)	176	(98.9)	
Equivocal (≥10.7 to <11.5)	1	(1.0)	1	(1.8)	0	(0.0)	2	(1.1)	
Positive (≥11.5)	0	(0.0)	0	(0.0)	0	(0.0)	0		
**HER2 testing by IHC assay**, *n* (%)									0.922
Number of non-missing	93		52		16		161		
Negative (0, 1+)	73	(78.5)	40	(76.9)	13	(81.2)	126	(78.3)	
Equivocal (2+)	20	(21.5)	12	(23.1)	3	(18.8)	35	(21.7)	
Positive (3+)	0	(0.0)	0	(0.0)	0	(0.0)	0	(0.0)	
**HER2 testing by FISH assay**, *n* (%)									0.786
Not amplified (gene copy number <4.0 or ratio <1.8)	71	(67.0)	41	(71.9)	12	(66.7)	124	(68.5)	
Equivocal (gene copy number 4.0–6.0 or ratio 1.8–2.2)	2	(1.9)	0	(0.0)	0	(0.0)	2	(1.1)	
Amplified (gene copy number >6.0 or ratio >2.2)	0	(0.0)	0	(0.0)	0	(0.0)	0	(0.0)	
Not performed	33	(31.1)	16	(28.1)	6	(33.3)	55	(30.4)	
**ER testing by Onco*type* DX ER score**, *n* (%)									0.072
Negative (<6.5)	0	(0.0)	1	(1.8)	1	(5.6)	2	(1.1)	
Positive (≥6.5)	106	(100.0)	56	(98.2)	17	(94.4)	179	(98.9)	
**Interpretation of ER testing by IHC assay**, *n* (%)									N/A
Negative (<1%)	0	(0.0)	0	(0.0)	0	(0.0)	0	(0.0)	
Positive (≥1%)	106	(100.0)	57	(100.0)	18	(100.0)	181	(100.0)	
**ER testing: % quantitation**, mean (SD) [median; range]	98	88.8 (17.3) [95; 9–100]	53	86.9 (20.4) [95; 11–100]	15	83.8 (24.2) [92; 3–100]	166	87.7 (18.9) [95; 3–100]	0.595
**PR testing by Onco*type* DX PR score**, *n* (%)									<0.001
Negative (<5.5)	1	(0.9)	10	(17.5)	13	(72.2)	24	(13.3)	
Positive (≥5.5)	105	(99.1)	47	(82.5)	5	(27.8)	157	(86.7)	
**Interpretation of PR testing by IHC assay**, *n* (%)									<0.001
Negative (<1%)	3	(2.8)	5	(8.8)	7	(38.9)	15	(8.3)	
Positive (≥1%)	103	(97.2)	52	(91.2)	11	(61.1)	166	(91.7)	
**PgR testing: % quantitation**, mean (SD) [median; range]	95	78.3 (24.2) [90; 5–100]	48	60.0 (34.9) [73; 1–100]	9	45.1 (35.3) [35; 3–95]	152	70.6 (30.4) [87; 1–100]	<0.001

SLNB (sentinel lymph node biopsy); ALND (axillary lymph node dissection); SD (standard deviation); HER2 (Human Epidermal Growth Factor Receptor 2); IHC (immunohistochemistry); FISH (fluorescence *in situ* hybridization); ER (estrogen receptor); PR (progesterone receptor). ^a^ *p* < 0.05 for a statistically significant difference among the three Recurrence Score result groups.

Endocrine therapy was recommended in 91.2% (*n* = 165) of patients overall, and there was a statistically significant difference across Recurrence Score groups (*p* < 0.029), with a higher percent of patients receiving endocrine therapy in lower Recurrence Score groups. Chemotherapy was recommended in all 18 patients with high Recurrence Score results, compared to 57.9% (33/57) in the intermediate and 19.8% (21/106) in the low score group (*p* < 0.001 for both the comparison across all groups and between the low and high groups). In the low Recurrence Score group, 85 patients (80.2%) were recommended endocrine therapy alone, compared to 24 (42.1%) in the intermediate group, and no one in the high Recurrence Score group (*p* < 0.001) (Table 3).

Comparing our study sample with aggregate data from all pN1mi patients who had a Recurrence Score result reported in the US during the same time period revealed no differences in age (mean 60.7 years in the study sample *vs.* 59.2 overall), Recurrence Score result (mean 17.8 in the study sample *vs.* 17.5 overall), HER2 status (0% positive in the study sample *vs.* 0.8% overall), ER status (98.9% positive *vs.* 98.6%), and PR status by Onco*typ*e DX (86.7% positive *vs.* 86.2%) (*p* > 0.05 for all comparisons) (Table 4).

This multicenter medical record review study examined how the Recurrence Score results may be used to inform treatment plans in HER2(−)/ER+ pN1mi breast cancer. We found chemotherapy was recommended significantly more often to patients with a high Recurrence Score result than to those with a low score. Similarly, endocrine therapy without chemotherapy was never recommended to patients in the high Recurrence Score result group, but was recommended to more than 80% of those in the low score group. Patients in this study were similar to the larger group of patients who were tested with the assay nationwide during the same time period.

We did not collect information from patient medical records regarding how the Onco*type* DX assay results were used by physicians in determining the treatment recommendations for patients in this study. Based on our prior cognitive interviews with oncologists, the typical process of arriving at treatment recommendations with Recurrence Score information may include a review of the patient’s clinicopathologic factors, consultations with a multidisciplinary physician group (e.g., tumor board), individual physicians (e.g., trusted colleagues), and consideration of the patient’s preference. Our findings suggest that clinicians use the Recurrence Score result in determining treatment recommendations for patients with HER2(−)/ER+ pN1mi breast cancer, and that the Recurrence Score provides information beyond that provided by traditional clinical and pathologic data. When treated with tamoxifen alone, pN1mi patients have a low recurrence risk [11], and by forgoing chemotherapy in some patients with low scores, physicians may be avoiding unnecessary chemotherapy and its attendant adverse effects [12,13]. Although determining cost savings was not a goal of the study, and respondents were not queried about cost, it may be that clinicians also consider the economic savings associated with reducing unnecessary chemotherapy [14,15,16,17].

Advances in surgical and analytical techniques have resulted in increased frequency of diagnosis of pN1mi disease, but there is still a debate about its prognostic significance. Several studies have found no significant difference in overall and/or disease-free survival between patients with micrometastasis and those with node-negative disease [18,19,20], suggesting that the treatment of micrometastatic disease should be the same as node-negative disease. The distribution of Recurrence Score results observed in the current study is similar to the distribution seen in node-negative patients, providing support for the thesis that these patient groups are similar at the level of breast cancer gene expression. Other studies provide evidence that micrometastasic disease does indeed confer a worse prognosis [21,22] and that adjuvant therapy may improve disease-free survival [23]. If micrometastatic breast cancer behaves like node-positive disease, it may still be possible to eliminate chemotherapy in a lower risk group without worsening outcomes through the use of the Recurrence Score result [24].

This study was limited by the retrospective design. We were only able to compare proportions of patients recommended chemotherapy for the different Recurrence Score result categories, and not the change in treatment recommendations from before to after the Onco*type* DX result was available. While prospective data on this population are unlikely to be forthcoming given the sample sizes and period of follow up that would be required for meaningful outcomes, further long term evaluations will be assessed in an ongoing phase III randomized clinical trial examining survival outcomes in patients with 1–3 positive nodes, HER2(−)/ER+ breast cancer with Recurrence Score of ≤25 undergoing standard adjuvant endocrine therapy with or without chemotherapy [25]. Additionally, given the design of our study, we were unable to include a control group with treatment recommendations for patients with similar stage and type of breast cancer for whom treating physicians did not order the Onco*type* DX Recurrence Score. We also could not confirm whether distributions of treatment recommendations and the actual treatments administered differed in the study subjects since such data were not collected in this study, although we would expect such a difference, if any, would be minimal.

Other limitations include the collection of data at oncology centers chosen purposefully, rather than randomly. Because the study was designed to examine the impact of genomic testing on decision-making, it focused on oncologists who used the test, rather than on a random sample of oncology practices around the country. As a result, these findings should apply specifically to those patients for whom treatment decision-making was felt to require additional risk assessment. To confirm the comparability of the study subjects to a wider population, we compared the study sample to the entire patient population of HER2(−)/ER+ pN1mi breast cancer patients on whom the test was ordered during the same period. Patient demographics, Recurrence Score results, and pathologic features were all similar between the study group and the broader population. Detailed histopathologic data was not available for comparison. A final limitation is that the study included data on patients treated from 2007–2012, covering a period both before and after the test appeared in NCCN treatment guidelines. Our estimate of decision impact may therefore be conservative as clinicians may have been less likely to base treatment decisions on the results before the assay was included in guidelines [5,8].

Although our study has limitations, our results reveal important and novel information about the clinical utility of the Onco*type* DX Recurrence Score assay in HER2(−)/ER+ pN1mi breast cancer patients at oncology practices throughout the US. Additional key information about the Onco*type* DX assay will be revealed at the conclusion of the two prospective trials, the TAILORx (NCT00310180) and the RxPONDER (SWOG S1007; NCT01272037). The TAILORx trial aims to use the Onco*type* DX assay to determine which women (particularly those with the midrange Recurrence Score of 11–25) with early-stage breast cancer, whose tumors are ER-positive and/or PR-positive, HER2(−) and whose lymph nodes are negative, would be more likely to benefit from chemotherapy and reduce the use of chemotherapy in those who are unlikely to benefit from it. The RxPONDER trial, currently enrolling patients with node-positive, ER+, HER2-negative breast cancer who also have low to intermediate Onco*type* DX Recurrence Score results, aims to assess the benefit, if any, these patients get from the addition of chemotherapy to hormone therapy.

**Table 3:** Adjuvant treatment recommendations by Onco*type* DX Recurrence Score result.

	Recurrence Score <18 *n* = 106; 58.6%		Recurrence Score 18–30 *n* = 57; 31.5%		Recurrence Score ≥31 *n* = 18; 9.9%		Total *n* = 181		*p* value ^a^	*p* value ^b^
	Number	Estimate	Number	Estimate	Number	Estimate	Number	Estimate		
**Endocrine therapy**, *n* (%)	101	(95.3)	50	(87.7)	14	(77.8)	165	(91.2)	0.029	0.025
**Chemotherapy**, *n* (%)	21	(19.8)	33	(57.9)	18	(100.0)	72	(39.8)	<0.001	<0.001
**Treatment**, *n* (%)									<0.001	<0.001
Endocrine therapy without chemotherapy	85	(80.2)	24	(42.1)	0	(0.0)	109	(60.2)		
Chemotherapy without endocrine therapy	5	(4.7)	7	(12.3)	4	(22.2)	16	(8.8)		
Both endocrine therapy and chemotherapy	16	(15.1)	26	(45.6)	14	(77.8)	56	(30.9)		
**Patients with recommendation of chemotherapy**, *n* (%)	21		33		18		72			
**Chemotherapy regimen recommended**, *n* (%)									0.134	0.016
BOTH taxane- AND anthracycline-based	9	(42.9)	9	(27.3)	5	(27.8)	23	(31.9)		
Taxane-based, NO anthracycline	5	(23.8)	16	(48.5)	12	(66.7)	33	(45.8)		
Anthracycline-based, NO taxane	0	(0.0)	1	(3.0)	0	(0.0)	1	(1.4)		
Other	7	(33.3)	6	(18.2)	1	(5.6)	14	(19.4)		

^a^ *p* < 0.05 for a statistically significant difference among the three Recurrence Score result groups. ^b^ *p* < 0.05 for a statistically significant difference between Recurrence Score <18 and ≥31 groups.

**Table 4:** Comparison of study characteristics with Genomic Health data of pN1mi patients in a comparable time period.

	Study Data (*n* = 181)		Genomic Health Data ^a^ (*n* = 9328)		*p*-value ^b^
	Number	Estimate	Number	Estimate	
**Age** (years), mean (SD) [median; range]	60.7 (10.4) [60; 34–83]		59.2 (10.6) [60; 22–93]		0.055 ^c^
**Age**, *n* (%)					0.130
<50 years	26	(14.4)	1900	(20.4)	
50–64 years	92	(50.8)	4309	(46.2)	
≥65 years	63	(34.8)	3119	(33.4)	
**Onco*type* DXRecurrence Score result**, mean (SD) [median; range]	17.8 (8.8) [16; 0–50]		17.5 (9.8) [16; 0–100]		0.650 ^c^
**Onco*type* DX Recurrence Score result categories,** *n* (%)					0.536
<18 (low risk)	106	(58.6)	5643	(60.5)	
18–30 (intermediate risk)	57	(31.5)	2965	(31.8)	
≥31 (high risk)	18	(9.9)	720	(7.7)	
**HER2 testing by Onco*type* DX HER2 score**, *n* (%)					0.567 ^d^
Negative (<10.7)	176	(98.9)	9147	(98.1)	
Equivocal (≥10.7 to <11.5)	2	(1.1)	103	(1.1)	
Positive (≥11.5)	0	(0.0)	78	(0.8)	
**ER testing by Onco*type* DX ER score**, *n* (%)					0.778 ^d^
Negative (<6.5)	2	(1.1)	135	(1.4)	
Positive (≥6.5)	179	(98.9)	9193	(98.6)	
**PR testing by Onco*type* DXPR score**, *n* (%)					0.848
Negative (<5.5)	24	(13.3)	1283	(13.8)	
Positive (≥5.5)	157	(86.7)	8045	(86.2)	

SD (standard deviation); ER (estrogen receptor); PR (progesterone receptor). ^a^ Selection criteria for Genomic Health, Inc. Data: 1. ER Status specified on requisition = positive; 2. Nodal status = “Micromets”; 3. Tumor Type either “Ductal carcinoma, NOS”, “Lobular carcinoma, classic type”, “Lobular carcinoma, solid or alveolar type”, “Pleomorphic lobular carcinoma”, or “Invasive carcinoma, mixed pattern”; 4. Requisition date between 1 January 2007 and 31 December 2012; 5. Gender = Female; 6. World region = Domestic; 7. Patient age ≥18. (Note: Local HER2 status was not specified in the Genomic Health, Inc. data requisition.). ^b^ *p* < 0.05 for a statistically significant difference between the study sample and Genomic Health, Inc. data. ^c^ Comparison between means. ^d^ Pearson exact test.

## 4. Conclusions

Gene expression profiles are becoming an integral part of the personalization of cancer care. Clinicians who order the Onco*type* DX assay for their HER2(−)/ER+ pN1mi breast cancer patients use the results of the test to supplement clinical and pathologic information in making treatment recommendations. Our study shows that up to 80% of patients with low Recurrence Score results were recommended endocrine therapy alone compared to none of the patients with high scores resulting in a sparing of exposure to the side effects of chemotherapy in patients with little to no likelihood of benefit and providing the appropriate treatment with chemotherapy to the patients with the highest risk of distant recurrence.
